# Cell-Based Therapies for Glaucoma

**DOI:** 10.1167/tvst.12.7.23

**Published:** 2023-07-26

**Authors:** Joshua Luis, Karen Eastlake, William D. B. Lamb, G. Astrid Limb, Hari Jayaram, Peng T. Khaw

**Affiliations:** 1 NIHR Biomedical Research Centre for Ophthalmology, UCL Institute of Ophthalmology & Moorfields Eye Hospital, London, UK

**Keywords:** glial cell, glaucoma, stem cell

## Abstract

Glaucomatous optic neuropathy (GON) is the major cause of irreversible visual loss worldwide and can result from a range of disease etiologies. The defining features of GON are retinal ganglion cell (RGC) degeneration and characteristic cupping of the optic nerve head (ONH) due to tissue remodeling, while intraocular pressure remains the only modifiable GON risk factor currently targeted by approved clinical treatment strategies. Efforts to understand the mechanisms that allow species such as the zebrafish to regenerate their retinal cells have greatly increased our understanding of regenerative signaling pathways. However, proper integration within the retina and projection to the brain by the newly regenerated neuronal cells remain major hurdles. Meanwhile, a range of methods for in vitro differentiation have been developed to derive retinal cells from a variety of cell sources, including embryonic and induced pluripotent stem cells. More recently, there has been growing interest in the implantation of glial cells as well as cell-derived products, including neurotrophins, microRNA, and extracellular vesicles, to provide functional support to vulnerable structures such as RGC axons and the ONH. These approaches offer the advantage of not relying upon the replacement of degenerated cells and potentially targeting earlier stages of disease pathogenesis. In order to translate these techniques into clinical practice, appropriate cell sourcing, robust differentiation protocols, and accurate implantation methods are crucial to the success of cell-based therapy in glaucoma.

**Translational Relevance:** Cell-based therapies for glaucoma currently under active development include the induction of endogenous regeneration, implantation of exogenously derived retinal cells, and utilization of cell-derived products to provide functional support.

## Glaucoma Pathogenesis

Glaucoma is the major cause of irreversible visual loss worldwide, second only to the reversible causes of uncorrected refractive error and cataract.^1^ A variety of clinical presentations and disease etiologies can ultimately lead to glaucomatous optic neuropathy (GON), characterized by degeneration of retinal ganglion cells (RGCs) and optic nerve head (ONH) remodeling. In addition to these two disease-defining processes, intraocular pressure (IOP) is the major modifiable risk factor in the management of glaucoma, although a significant proportion of patients with glaucoma present with IOP values within the normal range.^2^ Understanding the causal relationships between IOP, ONH remodeling, and RGC degeneration in the etiology of glaucoma is key to the formulation of successful treatment strategies.

Cross-sectional studies have demonstrated IOP within the general population to follow an approximately Gaussian distribution with an exaggerated central peak and modest skew toward higher pressures.^3^^,^^4^ The original suggestion that glaucoma can be defined simply by an IOP of 21 mm Hg or higher has been fundamentally refuted.^5^ In modern glaucoma research, there is agreement across different study cohorts that using IOP values alone to diagnose glaucoma offers poor sensitivity and specificity.^3^^,^^6^ This diagnostic challenge is further compounded by the finding that glaucoma prevalence varies significantly between different regions and ethnic groups.^7^ Nevertheless, medical, laser, and surgical approaches to reduce IOP are currently the only approved clinical treatment strategies in the treatment of glaucoma.^8^

**Figure 1.**
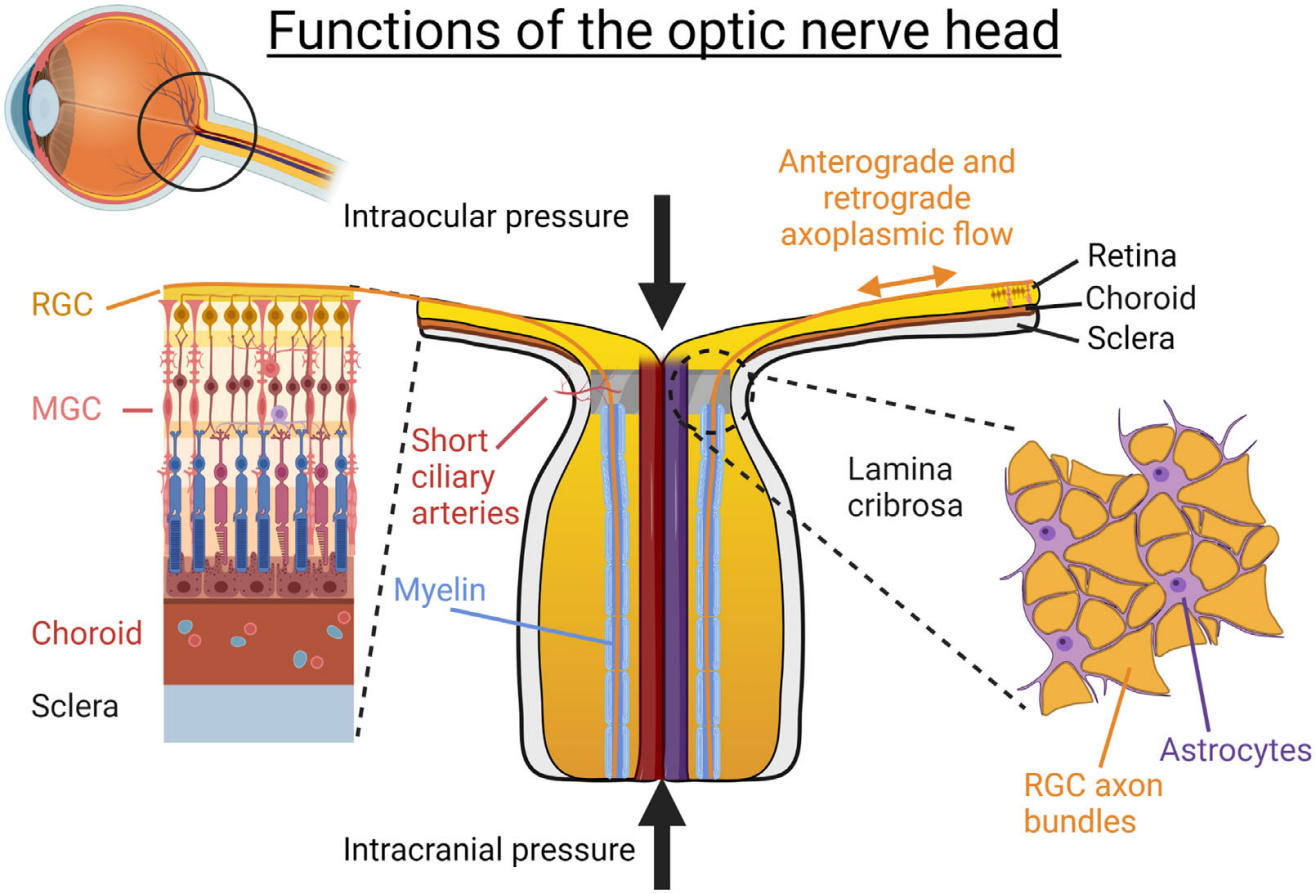
Normal physiologic function within the ONH. Complex biomechanical forces converge on the ONH, which results in a pressure gradient of around 3.5 mm Hg per 100 µm across the lamina cribrosa. The appropriate distribution of these forces, as well as structural and metabolic support from the astrocytes, allows for adequate flow within the short ciliary arteries and retinal ganglion cell axons.

Among the pathogenic features of glaucoma, characteristic cupping of the ONH as a result of tissue remodeling appears to be the most specific. In a healthy retina, over 1 million RGC axons converge at the ONH before turning sharply to exit the globe via the optic nerve. Prior to passing through the lamina cribrosa, RGC axons have very high energy requirements, as evidenced by the elevated concentration of cytochrome C oxidase and mitochondria seen in the prelaminar and lamina cribrosa regions.^9^^,^^10^ This decreases dramatically as the RGC axons acquire myelin sheaths after passing through the lamina cribrosa (Fig. 1).

The ONH is constantly subject to static and dynamic biomechanical forces, in part determined by IOP in its anterior aspect and intracranial pressure (ICP) in its posterior aspect.^11^ In a healthy individual, this creates a translaminar pressure gradient of around 3.5 mm Hg per 100 µm, which appears to cause a congestive effect to RGC axons even during physiologic conditions.^12^ The translaminar pressure gradient is particularly sensitive to postural changes, diurnal variation, and disease states where IOP may be increased or ICP decreased.^13^ Significant increases in the translaminar pressure gradient are associated with GON, as it compromises axonal transport through the lamina cribrosa.

The lamina cribrosa is a specialized porous collagenous plate, which provides structural integrity to the ONH. Situated between RGC axons and the lamina cribrosa are astrocytes, the major glial cell type found in this region, which provide structural and metabolic support to the RGCs.^14^^,^^15^ Astrocytes are intimately coupled to adjacent RGCs, blood vessels, and extracellular matrices to facilitate the distribution of key molecules such as neurotrophic factors^16^ and assist in the normal turnover and migration of mitochondria.^12^^,^^17^ There is also evidence that astrocytes are capable of remodeling the collagenous lamina cribrosa over time.^18^^,^^19^

At times of excess pressure-induced stress, studies have demonstrated ONH astrocytes to become reactive and decrease their expression of glial fibrillary acidic protein, a finding that consistently coincides with morphologic changes, axonal transport delay, and astrocyte swelling.^20^ A detailed electron microscopy study of the optic nerve after experimental induction of raised intraocular pressure demonstrated early dissociation between the astrocytes and their adjacent nerve fibers, as well as focal areas of axoplasmic holdup with potential deprivation of neurometabolic support to these highly energy-dependent nonmyelinated axons.^20^ This unfavorable sequence of events ultimately results in longer term antero- and retrograde degeneration, which forms the basis of the “energy theory” of glaucomatous damage.^21^

Another important aspect of the ONH structure is the blood supply to this region. Indeed, impaired blood flow is currently thought to be an important pathogenic factor for normal-tension glaucoma (NTG).^22^ This may be due to the observation that short ciliary arteries that supply the ONH appear to be particularly vulnerable to changes in ocular perfusion pressure, defined as the difference between systemic blood pressure and IOP,^23^ when compared to the central retinal artery. It should be noted that IOP reduction remains an effective treatment strategy in a large proportion of patients with NTG.

Maintenance of healthy RGCs requires the availability of essential metabolic substrates, specific trophic factors, and prompt removal of environment stressors.^24^ In particular, the neurotrophin family of proteins is crucial in promoting the survival of RGCs, while the BCL-2 family of genes exerts antiapoptotic effects.^25^ In glaucoma models, significant disruption of these pathways causes downstream activation of effector caspases and results in RGC death via apoptosis. Moreover, studies have increasingly revealed important roles played by other constituents of the neuroretina, including Müller glial cells (MGCs), microglia, and the complement cascade.^26^^,^^27^

**Figure 2.**
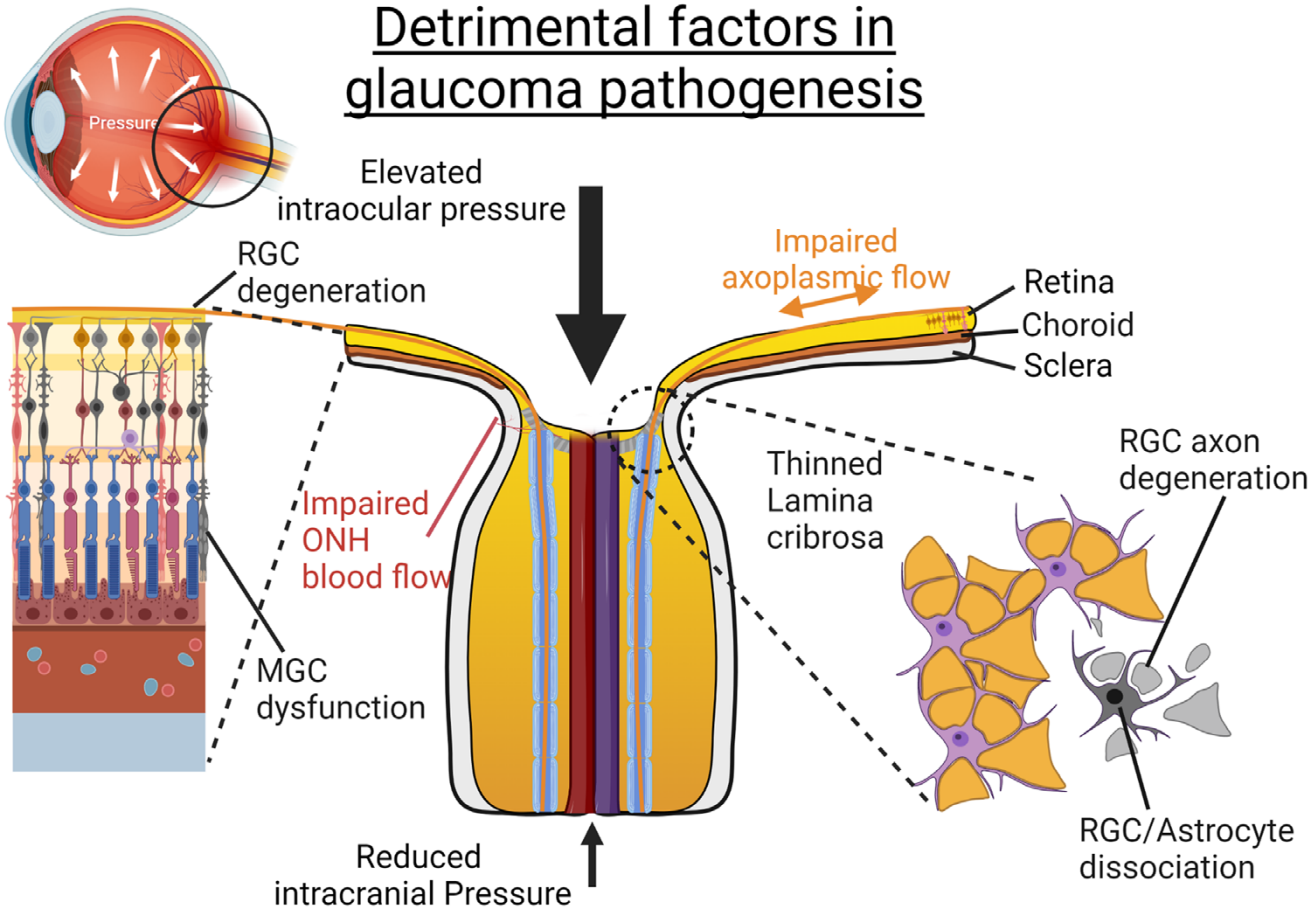
Detrimental factors in glaucoma pathogenesis. An increased pressure gradient across the lamina cribrosa can result from elevated intraocular pressure, reduced intracranial pressure, or abnormal tissue hysteresis. Over time, a characteristic “cupped” optic disc appearance can develop, which can cause thinning and bowing of the lamina cribrosa, as well as dissociation of astrocytes. In turn, these changes can lead to degeneration of RGC axons and glial cell dysfunction.

In summary, potential sources of axonal injury are counterbalanced by a range of protective mechanisms within the healthy individual. However, eyes of individuals who are particularly susceptible to GON can become overwhelmed in the presence of significant detrimental factors (Fig. 2). While the contribution of the various pathogenic mechanisms outlined in this section may depend on the glaucoma subtype and patient factors, the pathways ultimately converge to cause degeneration of RGC axons and cell bodies and therefore irreversible loss of vision.

## Development of Cell-Based Therapies

**Figure 3.**
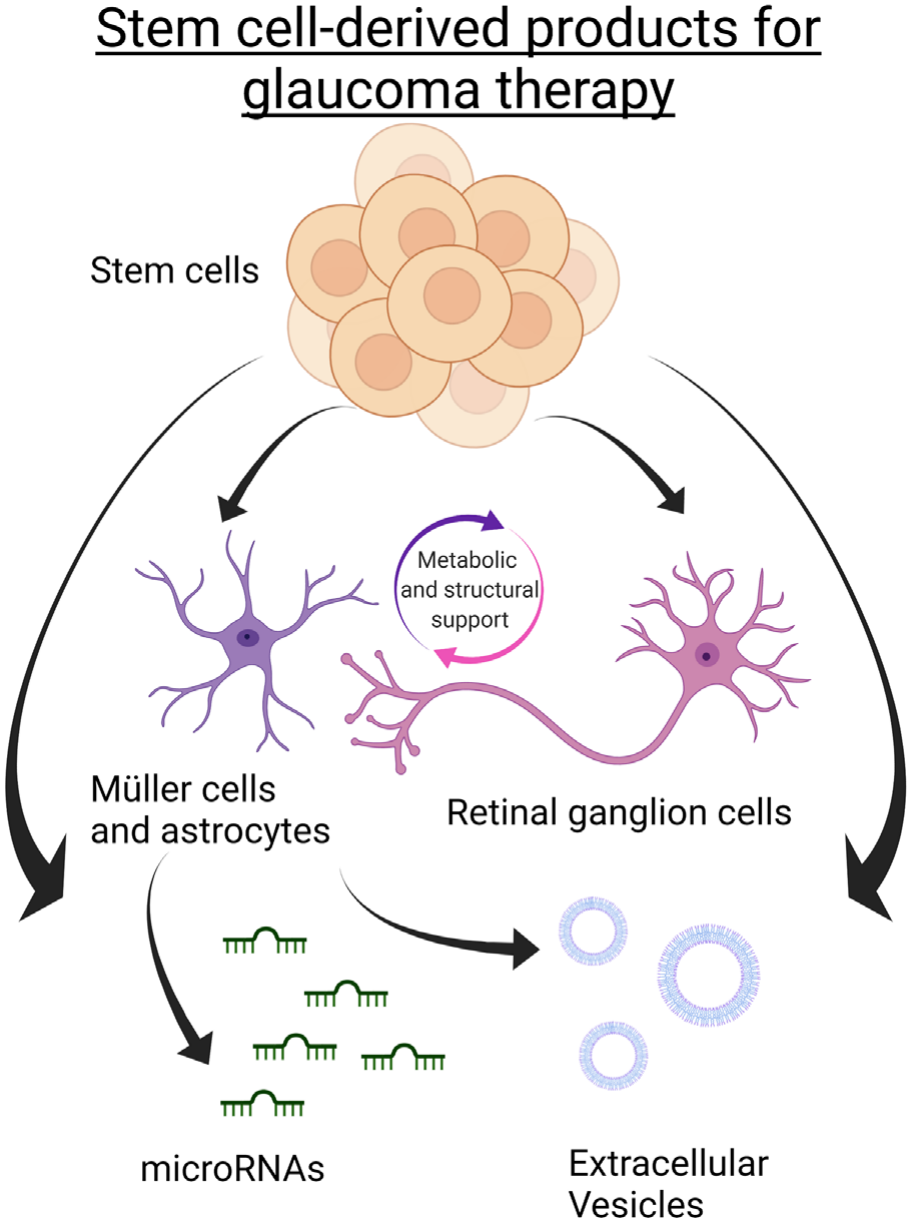
Stem cell–derived products for glaucoma therapy. Embryonic and induced pluripotent stem cells have been demonstrated to be capable of differentiating into Müller cells, astrocytes, and retinal ganglion cells in vitro. Animal experiments have demonstrated beneficial effects resulting from the direct implantation of these cells, while other recent studies have explored the potential therapeutic effects of products such as miRNAs and extracellular vesicles derived from various sources.

**Figure 4.**
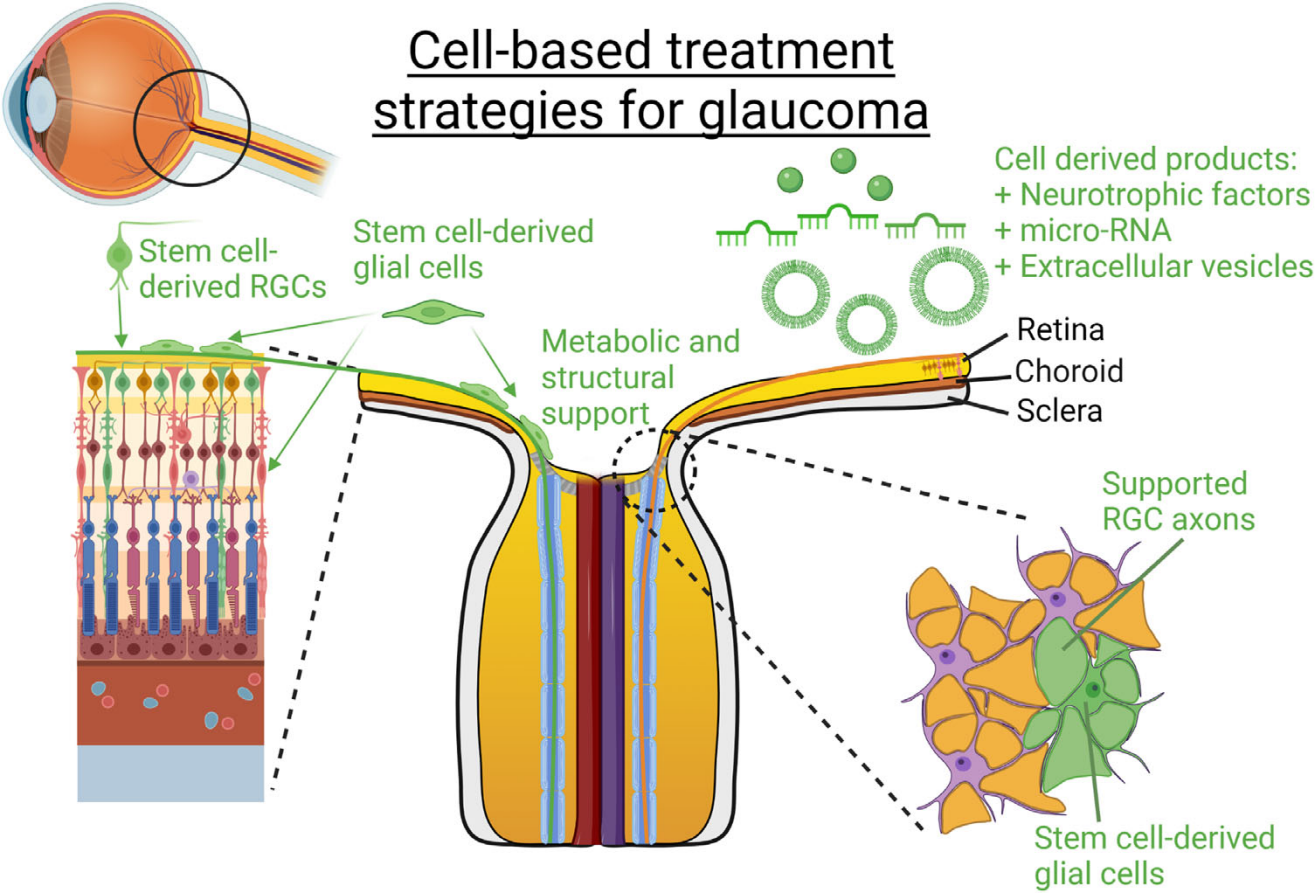
Treatment strategies for cell-based therapies in glaucoma. Cell replacement strategies aim to insert stem cell–derived glial cells and RGCs into their normal anatomic locations, whereas the implantation of glial cells into locations adjacent to RGCs has been shown to promote visual benefit. Furthermore, the injection of cell-derived products such as neurotrophic factors, miRNAs, and extracellular vesicles is a more recently developed modality of neuroprotection.

The retina is particularly well suited for the development of cell-based therapies for several reasons. First, most cell types within the retina are derived from a common neuronal lineage; second, the retina is arranged into well-defined layers that contain distinct cell types^28^; and third, in vivo visualization of the retina and its sublayers, up to a cellular resolution, is possible throughout any therapeutic period.^29^ A multitude of cell-based approaches have been employed with the goal of preventing and treating ocular diseases. Broadly speaking, these approaches can be divided into the induction of endogenous regeneration, replacement therapy using exogenous sources of cells, and application of cell-derived products for neuroprotection (Figs. 3 and 4).

### Endogenous Regeneration

Regeneration of the retina is a phenomenon that has been well described in certain species such as the zebrafish, where cell lineage tracing attributed the source of the regenerated cells to MGCs present in the inner nuclear layer of the central retina.^30^ Unfortunately, while a population of MGCs with neural stem cell characteristics has been identified in the human retina,^31^ retinal regeneration has not been observed to occur naturally in mammals. As such, considerable research has focused on identifying the fundamental mechanisms that allow for the repopulation of retinal cells.

A host of differentially expressed factors by zebrafish MGCs following injury has been found by both genetic and proteomic methods.^32^^,^^33^ Of particular importance are the presence of factors such as *stat3*, *ascl1*, and its downstream target *lin-28* in allowing the reentry of MGCs back into the cell cycle.^34^ Activation of these intracellular pathways is thought to be due, at least in part, to the paracrine release of cytokines, including tumor necrosis factor–α (TNF-α) and transforming growth factor–β (TGF-β),^35^ as well as growth factors such as epidermal growth factor (EGF) 1 and fibroblast growth factor (FGF).^36^

The detailed interactions of these pathways during the regenerative process have been extensively reviewed elsewhere.^37^ Although investigations in this area have substantially advanced our knowledge of intracellular mechanisms during retinal injury in different species, endogenous retinal regeneration has not yet been achieved in mammals. A major hurdle preventing the development of RGC replacement therapy is the sheer distance covered by the axons of RGCs during their projection to the brain. A typical RGC axon measures 50 mm in length, which is some 10,000 times the width of its cell body.^38^ In other words, if the diameter of an RGC axon (0.5–1 µm) was scaled up to the width of an average road, its projection would proportionally be 200 km. As such, precise methods of axonal guidance must be developed in order for such therapies to restore functional visual circuits between the retina and the brain.

### Cell Replacement Therapies

An alternative approach to treat degenerative retinal conditions is to prepare functional cells in vitro, followed by implantation into the damaged tissue. Although current technologies can derive therapeutic cells to closely match damaged cells, designing a method of delivery that promotes integration within the retinal circuit has proven to be exceptionally difficult.^39^ This is further complicated by the finding that evidence previously thought to demonstrate structural integration was in fact due to cytoplasmic material exchange.^40^^–^^42^ Nevertheless, several studies have demonstrated functional recovery following treatment despite a lack of integration of transplanted cells.^43^ Currently, a variety of approaches are being developed to achieve functional cell replacement. These can broadly be divided according to the type of source cell used (Fig. 3).

#### Embryonic Stem Cells and Induced Pluripotent Stem Cells

Embryonic stem cells (ESCs) are derived from the inner cell mass of the blastocyst and have the ability to differentiate to any cell type from the three germ layers, mesoderm, endoderm, and ectoderm.^44^ Discovered more recently, induced pluripotent stem cells (iPSCs) can be generated from adult cells by the overexpression of “Yamanaka factors,” which include Oct3/4, Sox2, Klf4, and cMyc.^45^^,^^46^ Various human fibroblasts, keratinocytes, and hematopoietic cells have been used to generate stable iPSC cell lines that show characteristics similar to ESCs.^47^^,^^48^

The use of human ESCs (hESCs) has been explored for use in a wide range of retinal degenerative conditions. Established protocols include the differentiation of RPE (retinal pigment epithelium) for AMD (age-related macular degeneration)-based therapies^49^ and the generation of retinal progenitors that express markers for differentiated rod and cone photoreceptors following injection to the rodent eye.^50^ More recently, studies have shown that ESCs can be differentiated to generate RGCs using either chemically defined or CRISPR-engineered protocols.^51^^–^^54^ Using a reporter stem cell line, RGCs expressing BRN3B and BRN3C can be FACS (fluorescence-activated cell sorting) sorted from adherent hESC-differentiated retinal tissue and grown on scaffolds to guide axonal outgrowth.^51^ These cells survive in culture and could be generated rapidly within 4 weeks, exhibited axonal outgrowth, and were viable when transplanted in adult rat retina.^52^

Both hESCs and human iPSCs (hiPSCs) have been used to generate three-dimensional retinal organoids that are shown to closely mimic in vivo development.^55^^–^^57^ This differentiation protocol developed by Nakano et al.^55^ has provided an easy-to-follow method for reliably generating laminated neuroretinal tissues. Adapted by many other laboratories,^56^^,^^58^^–^^60^ this novel strategy has paved the way for modern retinal research, including development, disease pathogenesis, and regenerative medicine. Retinal organoids are physiologically and metabolically functional, where photoreceptors^61^ and RGCs^62^ have been demonstrated to produce electrophysiologic responses to light activity. RNA sequencing analysis of retinal organoids has identified multiple RGC subtypes, which highlights their cellular diversity during in vivo development.^53^ One recent study demonstrated that RGCs derived from retinal organoids formed by hiPSCs survived for up to a month and improved function following injection into the vitreous of mice with optic neuropathy.^63^

Differentiation of photoreceptor precursors has also been increased in retinal organoids by the addition of COCO, an antagonist of the Wnt, Notch, TGF-β, and BMP pathways.^64^^,^^65^ The generation of RGCs using this method has proved more challenging, as there is a short competence window for the differentiation of RGCs during early retinal development. Furthermore, the number of RGCs decreases over time in retinal organoid cultures,^66^ while photoreceptor numbers increase.^56^ There is some in vivo evidence that the addition of microRNAs such as miR‐125b, miR‐9, and Let‐7 can increase the production of RGCs, in addition to promoting progenitor cell competence.^67^ Additionally, it may also be possible to regulate RGC differentiation by mitophagy-dependent metabolic reprogramming via glycolysis.^68^ Regulation of differentiation pathways such as Shh, TGFβ, Notch, and Wnt signaling has also shown to control RGC differentiation in mammalian retinas,^69^^–^^71^ while the addition of netrin-1 to retinal organoid culture medium has shown to enhance RGC neurite outgrowth.^72^ An in vitro approach using retinal organoids provides the perfect platform to discover and refine RGC differentiation techniques; as such, a robust protocol may be available in the not too distant future.

In some cases, methods to isolate cells from stem cell–derived retinal organoids may be more advantageous than trying to develop protocols to promote direct differentiation. RGCs are especially well suited for this, as they can be produced more rapidly due to being one of the first cell types to develop in retinal organoids.^72^ In summary, ESC- and iPSC-based methodologies are ideal for the development of cell-based disease models and therapies for glaucoma and other retinal diseases.

#### Mesenchymal Stem Cells

Mesenchymal stem cells (MSCs) are adult stem cells that can be derived from the bone marrow or umbilical cord. They are considered multipotent and have the capacity to differentiate toward cells of mesodermal lineage such as osteocytes, adipocytes, and chondrocytes.^73^ More recently, some studies have also shown that MSCs have the capacity to differentiate to neural lineages by the activation of Wnt/β-catenin, Notch, and Shh pathways^74^^–^^78^ and addition of growth factors, including EGF, bFGF (basic fibroblast growth factor), and HGF (hepatocyte growth factor).^79^^–^^81^ The secretome of MSCs comprise of a wide range of cytokines and growth factors, including interleukin (IL) 6, IL-8, BDNF (brain-derived neurotrophic factor), CNTF (ciliary neurotrophic factor), NGF (nerve growth factor), PDGF (Platelet-derived growth factor), LIF (leukemia inhibitory factor), NT-3 (eurotrophin-3), TGF-β2, and FGF2,^82^^–^^84^ and inflammatory factors, including TNF-α and IL-1β.^85^^,^^86^

MSCs have been extensively explored for their therapeutic use in the treatment of glaucoma. Intravitreal injections of MSCs in glaucoma-induced rodent eyes align along the ILM (Internal limiting membrane) and can survive for several weeks, resulting in increased RGC survival.^87^ A study compared the injection of human MSCs with their MSC-derived extracellular vesicles (EVs) into a rat optic nerve crush model, and the results showed sustained neuroprotection of RGCs by whole-cell treatment as compared to EVs alone, suggesting cellular-based therapies might confer better sustained neurotrophic support to the retina.^88^ MSCs have also been engineered to produce high levels of BDNF, and transplantation studies into hypertensive rat eyes demonstrated significant neuronal protection.^89^ Presently, few MSC-based therapies have reached clinical trial stages. Unfortunately, a recent study showed no significant changes in visual function as measured by ERG in two patients with open-angle glaucoma, and one patient was removed due to retinal detachment complications,^90^ which suggests that additional refinement is required for this type of cell therapy. Two further clinical trials using bone marrow–derived stem cells to treat optic nerve damage are currently in the recruitment phase (NCT01920867, NCT03011541).

### Müller Glial Cells

MGCs are the main glial cell type in the retina, as they span radially across all neuroretinal layers to form contacts with all cells of the neurovascular unit. The main functions of MGCs are to provide structural and metabolic support to retinal neurons similar to astrocytes within the ONH. MGCs become reactive in response to retinal injury, which can lead to the regeneration of lost neural cells in some species. Although this regenerative ability is not replicated in mammalian retina, MGCs with stem cell characteristics have been identified in the adult human eye. These cells can be isolated from postmortem tissues and spontaneously immortalize in vitro.^91^^,^^92^ Isolated human MGCs express characteristic stem cell markers such as SOX2, PAX6, βIII-tubulin, and notch 1 and, depending on culture supplementation, can be induced to become photoreceptor or retinal ganglion cell precursors.^93^^,^^94^

Extensive research has been conducted on the potential for MGCs in the treatment of glaucoma. In a rodent model of NMDA-induced retinal ganglion cell depletion, transplantation of human MGC-derived RGC precursors into the vitreous space resulted in a partial recovery of RGC function as judged by the negative scotopic threshold response of the electroretinogram.^93^ These cells align along the inner limiting membrane, but little to no integration is observed.^93^ Other studies have shown that rodent primary RGCs and progenitor-derived RGCs survive transplantation and become orientated along the host axons toward the ONH,^95^ but there has been limited evidence for substantial RGC integration and extension of axons through the ONH. One study found that following ablation of RGCs by intravitreal injection of NMDA to the feline eye, transplantation of MGCs into the intravitreal space resulted in partial recovery visual function.^43^ However, transplanted MGCs did not attach directly to the retina as seen in rodent studies but instead formed aggregates, suggesting that the vitreous may constitute a barrier for cell attachment onto the retina in the larger eye. As highlighted previously, RGC integration and axon projection remain major hurdles for this type of therapy.

Isolation of MGCs has been performed successfully from hiPSC-derived retinal organoids in vitro. Subsequent intravitreal transplantation of these MGCs into a rat model of RGC depletion has been demonstrated to induce a partial recovery of visual function.^60^ This study validates MGC implantation as a method of providing neuroprotection, and allogenic transplant studies are currently under way. There are important risks to consider when applying these cells to human therapies, however, such as the need for traceability, elimination of potential pathogens, and histocompatibility. Nevertheless, it is evident that MGCs are highly specialized to function within the retina while being a versatile source of cells for a variety of potential therapeutic applications.^96^

## Cell-Derived Products

As retinal regeneration has proven to be an immensely difficult goal, attention in recent years has shifted somewhat into strategies of neuroprotection. As we gain deeper understanding of the pathogenesis of glaucoma, various experimental approaches have emerged that aim to inhibit detrimental pathways or enhance endogenous protective mechanism to prevent GON.^97^ Although there is a large body of data to support the neuroprotective effects of neurotrophins and antioxidants, effective delivery into the eye remains a significant hurdle preventing the translation of our current knowledge into clinical practice.^25^

### Neurotrophins

The dependence of RGCs on neurotrophic factors is evident during optic nerve development,^98^ as well as in the adult tissue, where RGC survival is reliant upon the retrograde delivery of neurotrophic signals from targets within the central nervous system.^99^ In rodent models, adeno‐associated viral delivery of bFGF and BDNF can promote RGC survival following glutamate insult,^100^ while CNTF treatment significantly reduced RGC loss.^101^ There is also compelling evidence for RGC neuroprotection by the neurotrophin NGF.^102^^,^^103^

Despite these promising data, there remain concerns over the duration of any therapeutic effects from direct delivery of these neurotrophins, whereas a whole-cell transplant-based strategy may offer a longer-lasting solution. In support for this hypothesis, rat and human bone marrow–derived MSCs induced to secrete high levels of BDNF, GDNF (glial cell-derived neurotrophic factor), and VEGF (vascular endothelial growth factor) were found to exert a marked neuroprotective effect in a rodent optic nerve transection model following intravitreal injection.^104^ An alternative approach has incorporated the use of a polymeric capsule containing immortalized pigment epithelial cells transfected with the human CNTF gene.^105^ These devices have been demonstrated to be capable of sustained CNTF secretion and delivery to the posterior chamber for up to 1.5 years following implantation.^106^^,^^107^ Randomized phase I and II clinical trials are currently ongoing to evaluate the safety and efficacy of these devices in patients with primary open-angle glaucoma (NCT01408472, NCT02862938).

In order to realize the potential of neurotrophic factors in the future management of glaucoma, a safe, stable, and continuous system for their delivery remains a major challenge. Furthermore, despite deprivation of neurotrophic factors being characteristic of the glaucomatous retina, prolonged delivery of BDNF and TrkB receptor to axotomized optic nerve was unable to maintain long-term RGC survival.^108^ It is likely, therefore, that neurotrophin secretion constitutes a part but not all of the efficacy of whole-cell transplantation, as the roles of additional paracrine signals continue to be elucidated.

### MicroRNA and Extracellular Vesicles

MicroRNAs (miRNAs) are endogenous, small, noncoding RNAs. Their primary function is the posttranscriptional regulation of protein-coding gene expression by binding to the targeted messenger RNA (mRNA), which leads to the inhibition of translation or mRNA degradation. Through this system of interference, miRNAs have been demonstrated to play pivotal roles in cell proliferation, differentiation, and apoptosis.^109^^,^^110^ Several miRNAs have shown potential for RGC neuroprotection. These include miR-141-3p, which indirectly inhibits proapoptotic Bax and caspase 3 signaling pathways via targeting of docking protein 5 (DOK5)^111^; miR-93-5p, which was found to protect RGCs in culture from NMDA-induced cell death^112^; and miR-200a, which was found to preserve the thickness of the nerve fiber layer in a mouse model of glaucoma.^113^

In recent years, significant research interest in neuroprotective strategies has been focused upon the phosphatase and tensin homolog (PTEN) gene, the master inhibitor of the proregenerative mTOR/PI3K/Akt pathway.^114^ In nervous tissue, this pathway is responsible for the regulation of axon formation and extension during development, as well as the regeneration of peripheral nerve axons.^115^ Numerous miRNAs have been found to target PTEN and subsequently activate the mTOR (mammalian target of rapamycin) pathway, including miR-214,^116^ miR-1908,^117^ miR-494,^118^ and miR-21.^119^ In a model of glaucoma, inhibitors of miR-149 were shown to be neuroprotective of RGCs along with an associated upregulation of the PI3K/Akt pathway.^120^

Over the past decade, secreted EVs comprising cytosol enclosed in a lipid bilayer membrane have been identified as a major mode of intercellular communication,^121^ and some populations of EVs were found to be highly enriched in nucleic acids, particularly mRNAs and miRNAs.^122^ In the context of glaucoma, a recent study reported evidence of RGC axon regeneration using a heterogenous EV population isolated from the L-cell fibroblast line in an optic nerve crush model.^123^ Various MSC-derived EVs have also been found to significantly enhance survival of RGC and regenerate their axons, while partially preventing RGC axonal loss in rodent models.^124^^–^^127^ Furthermore, delivery of miRNAs via Schwann cell–derived exosomes into cultured neurons were found to promote neuritogenesis.^128^

Since EVs are capable of delivering their cargo directly into target cells, EVs derived from healthy cells may potentially have a comparable therapeutic potential as the cells themselves. Cell-derived EVs also contain a range of miRNAs to potentially activate multiple antiapoptotic and prosurvival pathways at once. As evidence accumulates for their neuroprotective efficacy, low immunogenicity, and high stability, EVs appear to be promising candidates as an adjunctive therapy to IOP-lowering medications and thus a potential future treatment for glaucoma.

## Conclusion and Future Directions

In recent years, there has been tremendous progress in the development of cell-based therapies for glaucoma. In addition to established strategies for endogenous regeneration and cell replacement, there is growing interest in more novel approaches using cell-derived products such as EVs. Appropriate cell sourcing, robust differentiation protocols, and accurate implantation techniques are all crucial to the development of successful cell-based therapy in glaucoma. In this rapidly progressing field, the increasing diversity of approaches and incremental refinements of existing techniques are bringing the prospect of clinical application ever closer to reality.
